# The Use of Complementary and Alternative Medicine Supplements of Potential Concern during Breast Cancer Chemotherapy

**DOI:** 10.1155/2016/4382687

**Published:** 2016-07-26

**Authors:** Erin Sweet, Fred Dowd, May Zhou, Leanna J. Standish, M. Robyn Andersen

**Affiliations:** ^1^Bastyr University, Kenmore, WA 98028, USA; ^2^Molecular Diagnostics Program, Fred Hutchinson Cancer Research Center, Seattle, WA 98109, USA; ^3^School of Public Health and Community Medicine, University of Washington, Seattle, WA 98195, USA

## Abstract

*Objective.* While many Complementary and Alternative Medicines (CAM) are unlikely to interact negatively with conventional oncology treatment, some ingestible CAM substances have biological activities that may reduce the effectiveness of chemotherapy or radiation. This study surveyed women with breast cancer in order to document the extent to which women with breast cancer use these CAM substances of concern concurrently with conventional treatments.* Methods.* A total of 398 women completed a survey describing their use of CAM at various time points in their cancer treatment. This report focuses on a subsample of 250 women receiving chemotherapy or radiation who reported using specific one or more of several chemotherapies.* Results.* Of those participating, 104 (43.7%) of those receiving chemotherapy (*n* = 238) and 45 (32.3%) of those receiving radiation (139; 58.4% of all patients) reported using one or more CAM substances that could be cause for concern when taken concurrently.* Conclusion.* Research is needed to understand the real risks associated with CAM and conventional polypharmacy. If risks associated with CAM conventional polypharmacy use prove to be substantial then improved systems to assure all women get advice regarding herb and supplement use during breast cancer treatment appear to be needed.

## 1. Introduction

Ingestible CAM substances, herbs, and other supplements are popular with the general public and among cancer patients. CAM use is reported by nearly half of all cancer patients [1] and studies suggest higher rates among women with breast cancer [2]. Although most CAM substances are safe for healthy people, the use of herbs and supplements during radiation treatment and chemotherapy is controversial. Herbs and supplements have known biological activities that could interact poorly with the activity or metabolism of many conventional cancer treatments and could theoretically reduce the effectiveness of conventional treatment or increase the toxicity associated with treatment. Review articles have highlighted potentially problematic polypharmacy interactions and suggested that many CAM substances should be considered contraindicated for use by cancer patients in active treatment with conventional chemotherapeutic agents [3–5]. Most conventional complementary treatment combinations of concern for cancer patients undergoing chemotherapy can be grouped into two categories. These include (1) antioxidants that may influence the action of radiation and some cytotoxic chemotherapies and (2) substances that may influence the pharmacodynamics of a chemotherapeutic regimen and thereby influence the effectiveness or safety of chemotherapy.

In more detail, concerns about CAM substance use during chemotherapy often focus on antioxidants because, while different antioxidants have different mechanisms of action, all reduce oxidative stress on tissues. Radiation treatments are thought to work in part by increasing oxidative stress in tumors and in theory their effectiveness would be diminished if a patient is also using a substance that reduces its prooxidant effect. Similarly, because both anthracyclines (e.g., doxorubicin/docetaxel/Taxotere) and platinum-based agents are intended to have their therapeutic effect through the generation of oxidative reactive oxygen species, there are reasons to be concerned that antioxidant use might reduce the effectiveness of these chemotherapies [5, 6]. On the other hand, Taxanes do not generate high levels of reactive oxygen species and thus for Taxanes use of antioxidants might be of less concern [7]. This theory-based concern is certainly warranted considering the basis for use of these treatments but may be excessive in the case on interactions between individual supplements and specific chemotherapy. Such evidence can only come from studies of specific interactions.

An example is the use of melatonin with chemotherapy and radiation. Melatonin functions as an antioxidant through several mechanisms including scavenging of free radicals, inducing of the expression of antioxidant enzymes, and reducing of the activation of prooxidant enzymes. This has raised concern about the concomitant use of melatonin with prooxidative therapies like chemotherapy and radiation during cancer treatment. To further complicate this issue, studies using cultured cells found that melatonin promoted the generation of reactive oxygen species at pharmacological concentrations in tumor cells thereby functioning as a prooxidant [8]. Even in tumor models unresponsive to melatonin alone, this hormone can significantly amplify the cytostatic and the cytotoxic effects triggered by other compounds or conventional drugs and reduce oxidative stress associated with chemotherapy, especially with anthracyclines, and radiotherapy [9, 10].

CAM substances can also interact with conventional chemotherapies through their effects on CYP pathways that influence drug metabolism. The most important CYP pathways for the metabolism of oncology pharmaceutical drugs are those involving the cytochrome P450 enzymes. The CYP cytochrome P450 3A4 isoform (commonly known as CYP3A4) is responsible for metabolizing greater than 50% of drugs which pass through the liver [11, 12]. Several herbs and other CAM substances influence CYP3A4's function by either inducing or inhibiting the actions of these enzymes and thus have the potential to influence the dose of a drug present in a patient's bloodstream [11, 12]. One study examining polypharmacy risks associated with CAM use during cancer treatment found that 43% of ovarian cancer patients take one or more of herb or supplement that could influence the effective dose of chemotherapy she receives. However, breast cancer patients are often put on different chemotherapeutic regimen than those with ovarian cancer so it is unclear how frequently CAM chemotherapy polypharmacy issues would be expected in a population of breast cancer patients.

The goal of this study was to determine the extent to which potentially dangerous combinations of substances including herbs and supplements were being used by breast cancer patients receiving chemotherapy. We also sought to determine how frequently herb and supplement use by patients was recommended by conventional physicians including an oncologist or primary care provider and CAM-providing naturopathic physicians.

## 2. Methods

### 2.1. Participants

This report describes a cross-sectional survey of a cohort of 398 women with breast cancer who received treatment in western Washington State, They include a convenience sample of women seeking integrative oncology (IO) treatment from local providers and a larger group of comparison women recruited from the local cancer registry. These women were selected based on similarity to the IO-seeking patients in their demographic characteristics and their stage of cancer at time of diagnosis. Women were eligible for the study if they spoke English fluently enough to complete surveys, were over the age of 21, had been diagnosed with breast cancer less than 2 years prior to their visit to the IO clinic, or were selected from the registry based on their similarity to an enrolled IO clinic patient. Clinic women were approached in person in the waiting room prior to either their first or second appointment with their IO provider. Women identified through the cancer registry were approached by mail in a two-step process, including a passive consent approach by the registry for release of personal data to researchers, and a later packet sent by our study. The packet contained a letter describing the study, two copies of the informed consent form, a medical records release, the enrollment questionnaire, and a self-addressed postage paid envelope in which to return their forms. Follow-up calls were made to women who did not respond to the initial mailing. Study methods and questionnaires for this research were reviewed by the IRBs of the Fred Hutchinson Cancer Research Center and Bastyr University.

### 2.2. Overview of Study Methods

The study questionnaire included sections assessing patient demographics (i.e., age, education, income, and race/ethnicity), conventional and CAM treatment, and a section assessing with whom patients consulted for advice about their use of herbs and supplements. Data was also collected from the cancer registry and medical records recording women's date and stage at diagnosis and the cancer treatments they received, including chemotherapies used and use of radiation. CAM substances of potential concern for potentially dangerous interactions were identified based on the scientific literature and clinical experience of naturopathic physicians board certified in naturopathic oncology.

### 2.3. Diagnosis and Treatments

Women were asked to indicate the year and stage of their cancer at initial diagnosis and the types of treatment they received. The information included the medications the patients received as part of their chemotherapy treatment(s). In order to assess CAM use before, during, and after conventional treatment for breast cancer, the questionnaire included a large chart listing many substances and describing different phases of conventional cancer treatment (initial surgery, first course of chemotherapy, radiation, after chemotherapy, and the time point when they completed the survey). Patients were asked to use this chart to indicate use of each of the almost 60 different herbs and other supplements throughout their treatment and following it. In all cases women were asked about their use of each substance by name. Where questions asked about substances that are also commonly ingested as foods (e.g., garlic, grapefruit juice, cranberry, and green tea) patients were asked to note substance use only if used in quantities greater than those associated with common cooking, particularly if they had consumed the substance in concentrated, dried, encapsulated, or powdered forms. This method of assessing use of CAM substances was used in a prior study [13]. We do not report on the use of all the CAM substances about which we asked in this report; this report focuses on a subset of the CAM substances which could be involved in unfortunate interactions with conventional therapy.

## 3. Results

Of the total sample of 398 breast cancer survivors enrolled, 350 reported having received either chemotherapy or radiation treatments the receipt of which could be confirmed by review of CSS records and dated prior to the date of completion of the enrollment survey. The remainder of this report focuses exclusively on that subsample of participating women. Of the 350, 68 (19.4%) presented with stage 3 or 4 disease, 147 (42%) presented with stage 2, 112 (32%) presented with stage 1, 3 (0.9%) presented with unknown stage, and 251 (71.7%) received radiation treatments. The most commonly prescribed chemotherapy agents were cyclophosphamide (IV or oral) 182 (52%), doxorubicin 105 (30%), docetaxel 101 (28.9%), and carboplatin 38 (10.8%); other drugs were used by 42 women (12%) of the sample. Of the women in this subsample, 89 (5.4%) received chemotherapy alone, 119 (34%) received radiation alone, and 132 (37.7%) received a combination of chemotherapy and radiation.  Table 1 shows the demographics of these chemotherapy or radiation using participants.

Table 2 summarizes the number of women who reported use of one or more of the antioxidants or herbs we analysed, during their chemotherapy and/or radiation treatment. Green tea (15.7%), melatonin (10.8%), vitamin C (11.4%), and vitamin E (10.6%) were the most commonly used antioxidants regardless of conventional therapy used. One hundred twenty-six (36%) of the women reported taking one or more form of antioxidant during chemotherapy and 77 (30.7%) reported the use of at least one antioxidant during radiation.

In calculating the percentage of women at potential risk for reduced effectiveness of their chemotherapy due to the use of antioxidants, only women using anthracyclines or platinum-based chemotherapy agents were considered potentially at risk because these chemotherapies have actions most dependent on oxidative effects. Overall 24 (22.9%) of women using doxorubicin and 15 (39.5%) of those using carboplatin were potentially at risk for reduced effectiveness of these medications because of their simultaneous use of antioxidants. Seventy-seven (30.7%) of women reporting that they received radiation also reported use of antioxidants at the time of their radiation treatment. As we noted in the introduction although melatonin is an antioxidant there are studies suggesting that it is, in fact, safe to use during chemotherapy and radiation. In this sample of 38 women, 25 (15%) of those using cyclophosphamide and 23 (9.2%) of those receiving radiation used melatonin.

For each CAM substance included in our survey, drug-CAM combinations which have the potential to affect the therapeutic efficacy of chemotherapy through modification of the activity of enzyme pathways that influence drug clearance were considered on a drug-by-drug basis. These assessments were based on their use of known CYP450 metabolism pathways. Docetaxel, for example, is metabolized by CYP isoforms 3A4, 3A5, and 2C8; thus blood levels of docetaxel might be affected by any of a list of herbs for which there is evidence that they influence these pathways. The list of potentially contraindicated herbs thus includes* Echinacea*, garlic, ginkgo, grapefruit juice, milk thistle, and St. John's wort. All of these substances influence CYP isoform 3A4 and are shown in Table 2. Sixty-eight (28.6%) of respondents used one or more CAM substances which affect CYP activity. The most commonly used substances in this category were green tea 33 (13.9%), vitamin E 23 (9.7%), garlic 18 (7.6%), and quercetin 7 (2.9%). The rates of use of one or more contraindicated CAM substance with the chemotherapy drugs we examined ranged from 0 (0%) (capecitabine, carboplatin methotrexate, vinorelbine, and 5-FU) to 53 (29.1%) (cyclophosphamide). Overall, considering women who used multiple chemotherapy drugs we found that 68 (28.6%) of our sample of women reported using at least one contraindicated CAM substance that could have affected the CYP metabolism of one or more of the chemotherapy drugs they reported using.

The CYP450 pathways required for the metabolism of one of the commonly used chemotherapies (i.e., carboplatin) are still unknown but are not believed to be metabolized via the CYP450 pathways. Therefore, no CAM substances were considered contraindicated for simultaneous use. As data are not available for all chemotherapies used in clinical practice, those who want to be conservative in their assessments might assume any or all CAM substances influencing hepatic metabolism have the potential to influence the metabolism of these agents.

Overall rates of use of conventional and CAM substance combinations of potential concern when used together during therapy for breast cancer varied by chemotherapy and ranged from 0 for capecitabine and vinorelbine to 67 (40.1%) for cyclophosphamide. When the course of radiation and chemotherapeutic treatment is considered as a whole and those at risk due to taking multiple chemotherapies, antioxidants, and CYP modifiers are considered, 104 (43.7%) of the 350 women of who received chemotherapy or radiation reported at least one CAM substance of concern in light of the chemotherapeutic regimen received.

All of the women in our sample were treated according to community standards by a conventional provider. Although some of our women were enrolled at a visit to an integrative oncology (IO) provider women were asked to complete their questionnaires prior to consultation with the IO provider and so were asked to provide information about CAM substances and CAM activities and CAM provider use sought prior to that visit. Of the women who reported CAM use during chemotherapy, 60 (42%) reported having had one or more of the CAM substances they used recommended or prescribed to them by a CAM provider while 34 (23.8%) reported receiving a recommendation for one or more of the CAM substances they used from a conventional provider.

Reported rates of recommendation of CAM substances by both conventional and CAM provider varied by substance. As illustrated in Table 3, the percentage of patients who reported that a conventional provider had recommended a specific antioxidant they were using ranged from 0% to 33%. In total, 27 (27.8%) of the 97 women who reported using antioxidants at the time of their chemotherapy reported having had at least one of their antioxidants they used recommended to them by a conventional provider. Rates of CAM provider recommendation for specific antioxidants ranged from 0% to 100%. In total, 54 of the 129 women who reported using antioxidant at the time of their chemotheraphy, whether or not the use would be considered contraindicated because of other therapies used, reported having at least one of their antioxidants recommended to them by a CAM provider.

Among the most popular CAM substances, 20 of the 60 women using green tea reported having had green tea recommended to them by a CAM provider and 4 a conventional provider, while 13 of the 37 women using vitamin C had it recommended to them by a CAM provider and 10 a conventional provider. As was noted earlier many of our women began using CAM supplements prior to diagnosis so the rates of recommendation/prescription may reflect recommendations offered by CAM or conventional providers prior to the cancer diagnosis and use of chemotherapy or radiation.

Levels of CAM use during chemotherapy or radiation were similar to levels of use reported before diagnosis with somewhat less than half of the sample reporting use at either occasion. But of the 169 (48.3%) of women who reported CAM use prior to diagnosis, only two-thirds of these (105; 62%) continued CAM use during chemotherapy. New use after diagnosis was also substantial of the 181 (51.7%) of women who reported no CAM use prior to diagnosis; 38 (21.0%) began using one or more CAM substances during chemotherapy (Table 4).

## 4. Discussion

Many herbs are available without prescription and may be used by women with breast cancer without discussion with any medical provider. Some foods with biological activity beyond their nutritive value (garlic, fruit juices with high antioxidant levels, and green tea) are also widely available in concentrated or dried forms for use as supplements. Review articles have suggested that some CAM substances should be considered contraindicated in combination with some chemotherapy drugs because either they act as antioxidants or they could influence liver metabolism of a chemotherapy drug. In this sample of breast cancer survivors we found that a substantial proportion 104 (43.7%) of women reported use of one or more substance potentially contraindicated given the chemotherapeutic agents they were receiving and 77 (30.7%) reported using antioxidants during radiation treatment. Women reported consulting with a conventional or CAM provider about only a small percentage of the substances they used and it is unclear if they consulted with anyone about the specific interactions of the CAM substances they were using after diagnosis with breast cancer.

However, this is not to say that these women are suffering ill-effects due to their CAM use, but only that these women may be at risk for such effects. Specific combinations of chemotherapy and antioxidants have been studied and there is evidence of survival benefit (e.g., melatonin) including reduction in side effects for some without diminished efficacy of the chemotherapy involved [14–18]. Most data suggest a synergistic effect with most high-dose dietary antioxidants and chemotherapy, and none have shown evidence of harm or lack of conventional treatment efficacy [19]. A systematic review of randomized controlled trials (RCTs) of melatonin in solid tumor cancer patients observed its effect on tumor remission, 1-year survival, and side effects due to radiochemotherapy. Eight eligible RCTs (*n* = 761), all of which studied solid tumor cancers, were included. The dosage of melatonin used in the 8 included RCTs was 20 mg orally, once a day. Melatonin significantly improved the complete and partial remission (16.5 versus 32.6%; RR = 1.95, 95% CI, 1.49–2.54; *P* < 0.00001) as well as 1-year survival rate (28.4 versus 52.2%; RR = 1.90; 95% CI, 1.28–2.83; *P* = 0.001) and dramatically decreased radiochemotherapy-related side effects including thrombocytopenia (19.7 versus 2.2%; RR = 0.13; 95% CI, 0.06–0.28; *P* < 0.00001), neurotoxicity (15.2 versus 2.5%; RR = 0.19; 95% CI, 0.09–0.40; *P* < 0.0001), and fatigue (49.1 versus 17.2%; RR = 0.37; 95% CI, 0.28–0.48; *P* < 0.00001). Effects were consistent across different types of cancer. No severe adverse events were reported [16]. Several other antioxidants, including ascorbic acid, may show the same dose-dependent functional relationship [20]. Similarly, there are several studies documenting the effectiveness of CAM use by cancer patients to manage treatment-related of side effects (i.e., alpha-lipoic acid to prevent peripheral neuropathy and CoQ10 to prevent cardiotoxicity) with evidence of risk only from in vitro studies [6, 21, 22]. However, this is a controversial area and there is a clear theoretical potential for interaction which may result in reduced treatment efficacy or increased toxicity.

A limitation of this study is that the questionnaire used did not ask women to indicate the doses of the substances taken. However, doses of CAM substances necessary to influence CYP450 enzyme activity and thus to modulate therapeutic doses of affected chemotherapies have often been studied only in vitro. Doses required for a modulator effect in humans are unknown; thus it is not known if patients took quantities significant enough to affect the metabolism of their chemotherapy drugs. Additionally, the active constituents present in CAM substances vary widely due to lack of standardization. Differences in cultivation and preparation alone are enough to create enormous variation in quantities of the active constituents. These variations are then multiplied by the many forms available (tea, standardized extract, ethanolic extract, solid extract, and whole herb) to patients. As many patients self-prescribe these substances without consultation and oversight from a physician specializing in these therapies, dose estimation is difficult if not impossible. Further research is needed to understand when combinations of CAM and conventional therapies of theoretical concern for concurrent use result in significant clinical effects that compromise the effectiveness of conventional therapy.

Another limitation of this study is that we did not ask of women why they were taking the substances. Many surveys of cancer patients have asked similar questions about CAM use and found that few cancer patients use CAM supplements as a treatment for their cancer with curative intent. Most users report an intention to improve immune function or their general health [2, 23, 24]. The most frequently reported substances found in this study suggest similar motives in this group. Green tea, vitamin D, and vitamin C are not widely promoted as cancer cures and are probably being taken for their perceived effectiveness in improving general health. Support for this conjecture also comes from noting the frequency with which women used CAM substances prior to diagnosis and during their initial treatment. The fact that the CAM substances are used with equal frequency at before diagnosis and during chemotherapy suggests that a significant percentage of women are not using them as a response to their cancer diagnosis.

With a substantial percentage of breast cancer patients using herbs and supplements there appears to be an urgent need for more research into the potential for these substance to interact negatively with conventional chemotherapy drugs. There is also a need to provide patients and their providers with better information on which to base decisions about the need to discontinue use of herbs and supplements during cancer treatment.

## Figures and Tables

**Table 1:** Patient characteristics (*N* = 350).

	*N*	%
*Racial background*		
Black or African American	3	0.9
White	317	90.6
Asian	7	2.0
Other	8	2.3
More than one race	2	0.6
Unknown/not reported	13	3.7

*Ethnicity*		
Non-Spanish, non-Hispanic	252	72.0
Mexican, south or central American	5	1.4
Other Spanish origin (includes European)	21	6.0
Unknown/not reported	72	20.6

*Current marital or partnership status*		
Married	255	72.8
Divorced	38	10.8
Single	25	7.1
Domestic partnership with opposite sex	20	5.7
Did not answer	8	2.3
Domestic partnership with same sex	4	1.1

*Income*		
No income	24	6.8
Less than $25,000/year	34	9.7
$25,000–50,000/year	63	18.0
$50,000–100,000/year	126	36.0
More than $100,000/year	86	24.6
Did not answer	17	4.8

*Stage at dx*		
Stage 0	20	5.7
Stage I	112	32.0
Stage II	147	42.0
Stage III	61	17.4
Stage IV	7	2.0
Unknown	3	0.9

*Conventional treatments received at time of BLQQ*		
Surgery	337	96.3
Chemotherapy	221	63.1
Radiation	251	71.7
Chemotherapy without radiation	89	25.4
Radiation without chemotherapy	119	34.0
Both radiation and chemotherapy	132	37.7

*Type of chemotherapy received at time of BLQQ* ^*∗*^		
Capecitabine	5	1.4
Carboplatin	38	10.8
Cyclophosphamide, IV	167	47.7
Cyclophosphamide, oral	15	4.3
Docetaxel	101	28.9
Doxorubicin	105	30.0
Lapatinib	3	0.9
Methotrexate	14	4.0
Vinorelbine	4	1.1
5-FU	16	4.6

*CAM treatment received*		
CAM substance use	235	67.1
Consultation with CAM provider	134	38.3

Ppts are those women that had begun chemotherapy or radiation by the time of their BLQQ, according to CSS and abstracted medical records.

Racial background, ethnicity, current marital or partnership status, and income are taken from the baseline QQ. Stage and conventional treatments are taken from CSS. Type of chemotherapy received is taken from ppt medical record abstraction.

^*∗*^Numbers for type of chemotherapy ≥350 with ≥1 chemo per woman.

CAM provider includes ND, Chinese medicine, massage, and chiropractor.

**Table 2:** CAM during chemotherapy (ref. natural medicines comprehensive database) *N* = 350.

	Capecitabine	Carboplatin	Cyclophosphamide (IV)	Cyclophosphamide (PO)	Docetaxel	Doxorubicin	Lapatinib	Methotrexate	Vinorelbine	5-FU	Radiation	Total
	*N* = 5	*N* = 38	*N* = 167	*N* = 15	*N* = 101	*N* = 105	*N* = 3	*N* = 14	*N* = 4	*N* = 16	*N* = 251	*N* = 350
*Antioxidants*												
Alpha-lipoic acid	0 (0.0)	2 (5.3)	6 (3.6)	0 (0.0)	6 (5.9)	1 (1.0)	0 (0.0)	0 (0.0)	0 (0.0)	0 (0.0)	6 (2.4)	11 (3.1)
Beta carotene											3 (1.2)	3 (0.9)
CoQ10	0 (0.0)	7 (18.4)	12 (7.2)	0 (0.0)	9 (8.9)	9 (8.6)	1 (33.3)	0 (0.0)	0 (0.0)	0 (0.0)	13 (5.2)	27 (7.7)
Glutathione											1 (0.4)	1 (0.3)
Grape seed extract											1 (0.4)	1 (0.3)
Green tea			33 (19.8)	0 (0.0)				0 (0.0)			34 (13.5)	55 (15.7)
Lycopene											0 (0.0)	0 (0.0)
Melatonin			25 (15.0)	0 (0.0)							23 (9.2)	38 (10.8)
Pycnogenol			0 (0.0)	0 (0.0)							0 (0.0)	0 (0.0)
Resveratrol			2 (1.2)	0 (0.0)	2 (2.0)		0 (0.0)				1 (0.4)	3 (0.9)
Selenium											4 (1.6)	4 (1.1)
Turmeric			15 (9.0)	1 (6.7)	9 (8.9)	9 (8.6)	1 (33.3)		0 (0.0)		11 (4.4)	26 (7.4)
Vitamin A			9 (5.4)	2 (13.3)				2 (14.3)			15 (6.0)	20 (5.7)
Vitamin C	0 (0.0)	7 (18.4)	16 (9.6)	3 (20.0)	9 (8.9)	10 (9.5)	1 (33.3)	3 (21.4)	0 (0.0)	3 (18.8)	23 (9.2)	40 (11.4)
Vitamin E	0 (0.0)	7 (18.4)	16 (9.6)	2 (13.3)	12 (11.9)	8 (7.6)	0 (0.0)	2 (14.3)	0 (0.0)	2 (12.5)	21 (8.4)	37 (10.6)
*Any of the above antioxidants*	*0 (0.0)*	*15 (39.5)*	*64 (38.3)*	*4 (26.7)*	*28 (27.7)*	*24 (22.9)*	*1 (33.3)*	*3 (21.4)*	*0 (0.0)*	*3 (18.8)*	*77 (30.7)*	*126 (36.0)*

*CYP activity*												
Bitter orange			0 (0.0)	0 (0.0)	0 (0.0)	0 (0.0)	0 (0.0)	0 (0.0)	0 (0.0)			0 (0.0)
Black cohosh			3 (1.8)	0 (0.0)				0 (0.0)				3 (1.3)
Dandelion						2 (1.9)	1 (33.3)					3 (1.3)
Devil's claw			0 (0.0)	0 (0.0)	0 (0.0)	0 (0.0)	0 (0.0)		0 (0.0)			0 (0.0)
DHEA			0 (0.0)	0 (0.0)	0 (0.0)	0 (0.0)	0 (0.0)		0 (0.0)			0 (0.0)
*Echinacea*			1 (0.6)	1 (6.7)	0 (0.0)	1 (1.0)	0 (0.0)		0 (0.0)			2 (0.8)
Feverfew			0 (0.0)	0 (0.0)	0 (0.0)	0 (0.0)	0 (0.0)	0 (0.0)	0 (0.0)			0 (0.0)
Garlic					7 (6.9)	10 (9.5)	1 (33.3)	0 (0.0)	0 (0.0)			18 (7.6)
Genistein								0 (0.0)	0 (0.0)			0 (0.0)
*Ginkgo biloba*			2 (1.2)	0 (0.0)	1 (1.0)	1 (1.0)	0 (0.0)	0 (0.0)	0 (0.0)			2 (0.8)
Goldenseal			0 (0.0)	0 (0.0)	0 (0.0)	0 (0.0)	0 (0.0)	0 (0.0)	0 (0.0)			0 (0.0)
Grapefruit juice			1 (0.6)	0 (0.0)	1 (1.0)	1 (1.0)	0 (0.0)	0 (0.0)	0 (0.0)			2 (0.8)
Green tea			33 (19.8)	0 (0.0)				0 (0.0)				33 (13.9)
Guggul tree			0 (0.0)	0 (0.0)		0 (0.0)			0 (0.0)			0 (0.0)
Kava kava						0 (0.0)	0 (0.0)	0 (0.0)	0 (0.0)			0 (0.0)
Licorice			4 (2.4)	1 (6.7)		4 (3.8)	1 (33.3)		0 (0.0)			5 (2.1)
Milk thistle			3 (1.8)	0 (0.0)	3 (3.0)	1 (1.0)	1 (33.3)		0 (0.0)			5 (2.1)
Peppermint oil			3 (1.8)	0 (0.0)	2 (2.0)	0 (0.0)	0 (0.0)		0 (0.0)			3 (1.3)
Pycnogenol			0 (0.0)	0 (0.0)								0 (0.0)
Quercetin			6 (3.6)	0 (0.0)	1 (1.0)	5 (4.8)	1 (33.3)		0 (0.0)			7 (2.9)
Red clover			0 (0.0)	0 (0.0)	0 (0.0)	0 (0.0)	0 (0.0)		0 (0.0)			0 (0.0)
Resveratrol			2 (1.2)	0 (0.0)	2 (2.0)	1 (1.0)	0 (0.0)		0 (0.0)			3 (1.3)
*Schisandra*						0 (0.0)	0 (0.0)		0 (0.0)			0 (0.0)
St. John's Wort			1 (0.6)	0 (0.0)	0 (0.0)	1 (1.0)	0 (0.0)		0 (0.0)	0 (0.0)		1 (0.4)
Valerian			0 (0.0)	0 (0.0)	0 (0.0)	0 (0.0)	0 (0.0)		0 (0.0)			0 (0.0)
Vitamin E			16 (9.6)	2 (13.3)	12 (11.9)							23 (9.7)
*Any of the above CYP*	*0 (0.0)*	*0 (0.0)*	*50 (29.9)*	*3 (20.0)*	*23 (22.8)*	*22 (20.9)*	*2 (66.7)*	*0 (0.0)*	*0 (0.0)*	*0 (0.0)*		*68 (28.6)*

*Any of the above*	*0 (0.0)*	*15 (39.5)*	*67 (40.1)*	*5 (33.3)*	*35 (34.6)*	*36 (34.3)*	*2 (66.7)*	*3 (21.4)*	*0 (0.0)*	*3 (18.8)*	*77 (30.7)*	*104 (43.7)*

Total is chemotherapy patients who took at least one of the named chemo drugs or had radiation therapy at the time the baseline questionnaire was filled out.

**Table 3:** Discussed CAM use with CAM or conventional provider (*N* = 350).

Supplement	Used during chemo/radiation	Discussed with	
		CAM provider	Conventional provider
	*N*	*N* (%)	*N* (%)
*Antioxidants*			
Alpha-lipoic acid	11	6 (54.5)	2 (18.2)
Beta carotene	5	1 (20.0)	1 (20.0)
CoQ10	27	15 (55.5)	4 (14.8)
Glutathione	5	3 (60.0)	1 (20.0)
Grape seed extract	3	1 (33.3)	0 (0.0)
Green tea	60	20 (33.3)	4 (6.7)
Lycopene	1	1 (100.0)	0 (0.0)
Melatonin	44	29 (65.9)	7 (15.9)
Pycnogenol	0	0 (0.0)	0 (0.0)
Resveratrol	3	1 (33.3)	1 (33.3)
Selenium	7	3 (42.9)	1 (14.3)
Turmeric	26	14 (53.8)	2 (7.7)
Vitamin A	24	9 (37.5)	5 (20.8)
Vitamin C	40	13 (32.5)	8 (20.0)
Vitamin E	37	13 (35.1)	10 (27.0)

*Any antioxidant*	129	54 (41.9)	32 (24.8)

*CYP activity*			
Bitter orange	1	0 (0.0)	0 (0.0)
Black cohosh	5	2 (40.0)	2 (40.0)
Dandelion	4	2 (50.0)	0 (0.0)
Devil's claw	0	0 (0.0)	0 (0.0)
DHEA	1	1 (100.0)	0 (0.0)
*Echinacea*	4	2 (50.0)	0 (0.0)
Feverfew	0	0 (0.0)	0 (0.0)
Garlic	29	6 (20.7)	0 (0.0)
Genistein	2	1 (50.0)	0 (0.0)
*Ginkgo biloba*	2	1 (50.0)	1 (50.0)
Goldenseal	0	0 (0.0)	0 (0.0)
Grapefruit juice	2	1 (50.0)	0 (0.0)
Green tea	60	20 (33.3)	4 (6.7)
Guggul tree	0	0 (0.0)	0 (0.0)
Kava kava	1	1 (100.0)	0 (0.0)
Licorice	7	4 (57.1)	0 (0.0)
Milk thistle	8	3 (37.5)	1 (12.5)
Peppermint oil	4	1 (25.0)	1 (25.0)
Pycnogenol	0	0 (0.0)	0 (0.0)
Quercetin	8	6 (75.0)	2 (25.0)
Red clover	0	0 (0.0)	0 (0.0)
Resveratrol	3	1 (33.3)	1 (33.3)
*Schisandra*	0	0 (0.0)	0 (0.0)
St. John's Wort	2	0 (0.0)	0 (0.0)
Valerian	1	0 (0.0)	0 (0.0)
Vitamin E	37	13 (35.1)	10 (27.0)

*Any CYP*	109	38 (34.9)	18 (16.5)

*Any of the above*	143	60 (42.0)	34 (23.8)

**Table 4:** CAM use (*N* = 350).

Use before dx	Use during chemo/radiation		Total
	No	Yes	
No	143	38	181
Yes	64	105	169

Total	207	143	350

Ppts are those women that had begun chemotherapy or radiation by the time of their BLQQ, according to CSS and abstracted medical records. CAM use data is from BLQQ.
